# Poor Compliance to Hormone Therapy and Decreased Bone Mineral Density in Women with Premature Ovarian Insufficiency

**DOI:** 10.1371/journal.pone.0164638

**Published:** 2016-12-01

**Authors:** Anne Bachelot, Carole Nicolas, Solenne Gricourt, Jérôme Dulon, Monique Leban, Jean Louis Golmard, Philippe Touraine

**Affiliations:** 1 AP-HP, IE3M, Hôpital Pitié-Salpêtrière, Department of Endocrinology and Reproductive Medicine, Centre de Référence des Maladies Endocriniennes Rares de la Croissance, Centre de Référence des Pathologies Gynécologiques Rares, Paris, France; 2 Université Pierre et Marie Curie, Univ Paris, Paris, France; 3 AP-HP, Hôpital Pitié-Salpêtrière, Department of Hormonal Biochemistry, Paris, France; 4 AP-HP, Hôpital Pitié-Salpêtrière, Clinical Research Unit, Paris, France; Van Andel Institute, UNITED STATES

## Abstract

Premature ovarian insufficiency leads to through infertility and estrogen deficiency. Optimal management encompasses estrogen replacement therapy. Long-term outcome of women with POI is not known. We design a study to evaluate the medical care, hormone replacement therapy compliance and bone mineral density (BMD) in POI women with at least a five-year follow-up after the first evaluation. One hundred and sixty-two patients (37.3±8.0 years) were evaluated (follow-up 7.9±2.8 years). Sixty-nine patients (42.6%) had stopped their hormone replacement therapy (HRT) for at least one year during the follow up period. BMD determination at initial evaluation and at follow-up visit was completed in 92 patients. At first evaluation, 28 patients (30%) had osteopenia and 7 (8%) had osteoporosis. At follow up, 31 women (34%) had BMD impairment with osteopenia in 61% and osteoporosis in 5%. In univariate analysis and multivariate analysis, there was a significant loss of femoral BMD in women who had stopped their HRT for over a year. In conclusion, this first study concerning long-term follow-up of POI patients shows the poor compliance to their HRT, despite its importance in the prevention of bone demineralization. This study reinforces the need for follow up and specific care for POI women.

## Introduction

Premature ovarian insufficiency (POI) is a disorder affecting approximately 1% of women under 40 years of age [1]. POI encompasses a heterogeneous spectrum of conditions, through two major mechanisms: follicle dysfunction and follicle depletion [2]. Although causes such as autoimmunity, monosomy X and environmental factors play a role in POI, the aetiology in most cases remains unknown [3].

Besides impairing the quality of life through infertility and estrogen deficiency, the long-term outcome of these women is actually not known. Indeed, most women with POI experience sustained sex steroid deficiency for longer periods compared with women who experienced spontaneous menopause around middle age. The long-term effects of POI are extrapolated from studies on the age of menopause onset, the consequences of prophylactic bilateral oophorectomy or Turner syndrome [4–9]. These observational and cross-sectional studies are heterogeneous, and they report increased risk for osteoporosis, cardiovascular events and psychological effects with early age of menopause or Turner syndrome [4–9]. Optimal management of POI women encompasses estrogen deficiency compensation by estrogen replacement therapy, and management of the infertility, for which oocyte donation or adoption could be proposed. Some studies have shown that women with POI have lower bone mineral density [3,10,11]. Nevertheless, the outcome of these women in terms of medical care, hormonal replacement therapy compliance and bone health status is not known.

We therefore conducted a longitudinal study of POI women, with at least five years of follow up after their first evaluation. To investigate the evolution of this disorder, we assess the initial and follow up medical managment, bone health status and hormone replacement therapy (HRT) use of POI patients.

## Subjects and Methods

### Patients

A cohort of POI patients was set up in our department in 2001. As described in detail previously [12], it included idiopathic POI patients referred to our department since 1997, and included 542 consecutive patients in December 2013. The patients included in this cohort were previously described [3,12], as were their clinical, hormonal, ovarian morphology and bone mineral status [12]. Among the 542 patients, records were available for 507 patients; they represent our active cohort POI patients who will be considered latter in this study. Every woman with POI diagnosis was prescribed with hormonal substitution therapy, but for various reasons, some of them did not or stopped taking it.

**Fig 1:**
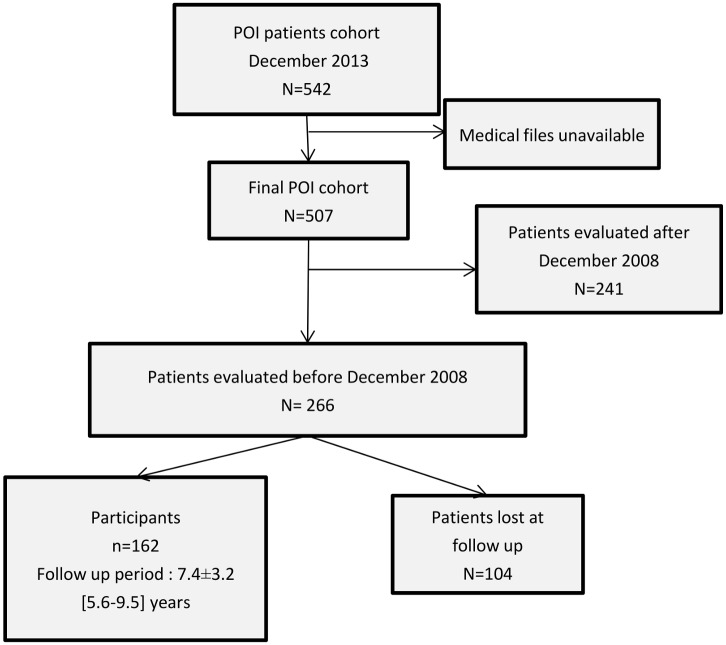
Flow chart of the POI patients included in the study.

We designed a cross-sectional study of patients with POI, at least five years after a first evaluation in our department (Fig 1). POI patients were contacted in order to evaluate their medical follow-up, HRT status and bone health status. Among the cohort of 507 patients, 266 patients were eligible to enter this study, i.e. they were all evaluated in our department before December 2008 (B1). These 266 patients were contacted for the present study. Forty-eight patients were regularly followed up in our department. The remaining patients were contacted by mail or phone. A second evaluation, called B2, was proposed for these patients, consisting in medical history recording, clinical evaluation including, fasting blood sampling for biological measurement (25 OH Vitamin D, PTH and TSH) and bone mineral density (BMD) assessment. Medical files concerning medical follow up, use of HRT, personal and familial reproductive history were recorded. Concerning the use of HRT, referring the medical files, patients who did not stop their HRT more than one year during follow up (excluding the period of pregnancy, either spontaneous or after oocyte donation) were considered as patients who never stopped their HRT. In France, retrospective studies and usual care study such as ours do not require institutional review board/institutional ethics committee approval to analyze the data and publish the results. An information note concerning the study had been commentated to all women and an oral informed consent of the patient had been obtained from all participants. Patients who did not wish to come to our department for this evaluation were given the option of answering by phone or mail. Bone mineral density was therefore performed outside of the hospital, using the same reference curves to calculate the women's T-scores. The data collected in the study are included in the CEntre de MAladies Rares (CEMARA) database, which has received approval from the CNIL.

### Hormone measurements

LH, FSH, Estradiol, inhibin B and AMH were measured as described in detail previously [12]. Serum AMH concentrations were measured in duplicate using AMH/MIS Elisa^®^ Immunotech, Beckman-Coulter from 2006 to 2009 and EIA AMH /MIS^®^ Immunotech, Beckman-Coulter from 2009 to 2014. Samples collected before 2006 were frozen at -20 C until assay. The intra- and interassay coefficients of variation for AMH determination were 5.3 and 8.7%, respectively. The AMH assay sensitivity was 0.05 μg/l. In women with normal menstrual cycles, the normal range for day 3–5 of the cycle is 2.2–6.8 μg/l. Plasma TSH was measured by immunofluorometric assay (Roche Diagnostics GmbH, Sandhofer Strasse 116, D-68305 Mannheim). The interassay coefficient of variation was 1.7 to 2.9%. The detection limit was 0.01 mIU/l. Plasma PTH was measured by immunofluorometric assay (Roche Diagnostics GmbH, Sandhofer Strasse 116, D-68305 Mannheim). The interassay coefficient of variation was 6.2 to 8.5%. The detection limit was 6 pg/ml. Plasma 25OH Vitamin D was measured by immunofluorometric assay (DiaSorin inc, 1951 Northwestern Ave, Stillwater,MN 55082 USA). The interassay coefficient of variation was 8.5 to 10.9%. The detection limit was 4 ng/ml.

### Bone assessment

Femoral neck, total hip and lumbar spine BMD were assessed by the operator using a Hologic Densitometer QDR 1000. The daily quality control showed a 0.52% CV during the performance of these tests. BMD results at the femoral neck, total hip, and lumbar spine (L_1_–L_4_) were evaluated and expressed as absolute values in g/cm^2^ and *T*-scores ((BMD–peak bone mass)/S.D). OFELY Caucasian reference curves were used to calculate the women's *T*-scores [13]. We were able to use *T*-scores to interpret and compare the results, since all our patients were under the age of 40 at initial evaluation and peak bone mass in the general population does not decrease at that age [14]. In accordance with World Health Organization criteria, osteoporosis was defined as a *T*-score <–2.5 S.D., and osteopenia was defined as a *T*-score between –2.5 and –1 S.D. Normal bone has a BMD *T*-score of –1 S.D. or higher [14].

### Statistical analysis

Descriptive statistics used means ±sd or median [interquartile interval] for quantitative variables and numbers (percentages) for qualitative ones. Comparisons between two groups were performed using Student’s t tests for quantitative variables and chi^2^ tests or Fisher’s exact tests for qualitative variables. Comparisons between three groups were performed using non parametric ANOVAs (Kruskal-Wallis tests) for quantitative variables and Fisher’s exact tests for qualitative variables, because validity conditions were not met for the chi^2^ test. Loss of vertebral and femoral BMD during follow up were analysed using multivariable linear model estimated by stepwise model selection. All computations were performed using the SAS V9.3 statistical package. All tests were two-sided, with a p-value < 0.05 considered as significant.

## Results

### Population

Two hundred and sixty six patients were eligible to participate to this study, and 162 agreed to take part (61%). Of these, 96 (59%) returned to our centre for a second evaluation and the remaining 66 answered the questionnaire by mail or phone. The remaining 104 patients were either lost at follow-up (n = 79, 76%), dead (n = 1) or declined the study (n = 24, 23%). These 104 patients did not differ from the 162 studied patients in term of clinical or hormonal presentation, except for the presence of autoimmunity markers at first evaluation (Table 1).

**Table 1:** Clinical and hormonal characteristics of the POI patients included (n = 162) or not (n = 104) in the study.

	Participants n = 162	Patients lost at follow up n = 104	p value
**Age at diagnosis (years)**	27.4 ± 7.9	27.9 ± 8	0.61
**Age at first evaluation (years)**	29.5 ± 7.8	30.3 ± 7.7	0.58
**BMI at first evaluation (kg/m**^**2**^**)**	22.91 ± 4.6	23.4 ± 4.7	0.44
**Tobacco use**	44/162 (27.2%)	27/74 (32.4%)	0.40
**All autoimmunity diseases**	30/162 (18.52%)	9/103 (8.7%)	0.028
**Familial history of POI**	36/161 (22.4%)	18/101 (17.8%)	0.37
**Secondary amenorrhea**	130/162 (80.2%)	86/103 (83.5%)	0.50
**POI with resumption**	55/162 (34.9%)	36/103 (35%)	0.8
**AMH at first evaluation (ng/ml)**	0.46 ± 0.2	0.31 ± 0.14	0.29
**FSH at first evaluation (IU/l)**	75.0 ± 42.6	73.8 ± 40.5	0.81
**Estradiol at first evaluation (pg/ml)**	27.7 ± 43.6	39.1 ± 97.3	0.27

Data are expressed as mean ±sd or number (%)

The first evaluation was performed 2.1±0.3 [0–3] years after POI diagnosis. The mean follow-up period was 7.4±3.2 [5.6–9.5] years. At baseline, the mean age at the time of the diagnosis for these 162 POI patients was 27.4±7.9 [20.0–33.7] years, the mean age at the first evaluation was 29.5±7.8 [23.2–36.0] years and the mean age at second evaluation was 37.3 ± 8.0 [30.7–44.0] years.

Among the 162 POI patients, 48 (30%) were actively followed up in our department; 64 (40%) were followed-up by their gynaecologist, 6 (4%) by an endocrinologist and 34 (20%) by their general practitioner. Absence of any medical follow-up was reported by 10 patients (6%).

At B2 evaluation, only 100 patients (61.7%) were under HRT. Among them, 3 used combined oral contraceptives, 50 oral estradiol associated with progestin and 37 transdermal estradiol associated with progestin. In the remaining 10 patients, the type of HRT was not informed.

During the follow up period, 69 patients (42.6%) have stopped their HRT at least one year outside of pregnancy. Of these, 10 had a contraindication for the use of estrogens: 2 had a personal history of breast cancer at 47 and 39 years (this patient had also familial history of breast cancer), occurring 9 and 3 years respectively after POI diagnosis; 5 for cardiovascular disease and 3 for thromboembolic disease. Fourteen women had never received hormone replacement therapy since diagnosis. The main reason for discontinuation of HRT was the reporting by these women of an absence of subjective benefice, i.e. quality of life or fertility improvements (n = 32.6%). Other reasons were the presence of diverse side effects (n = 9, 18%), the fear of breast cancer (n = 7, 14%) and weariness (n = 2, 4%). Depending on the practitioner providing monitoring of the POI, HRT was stopped in 10 patients (90%) in the absence of medical follow-up, in 14 patients followed in our department (30%), including 4 with a contraindication, and in 18 patients followed by their general practitioner (53%) Finally, treatment was also stopped in 24 women followed up by their gynaecologist (39%) and 3 (43%) patients followed by an endocrinologist. The comparison between women who stopped their HRT for more or less than one year is represented in Table 2: women who have stopped their HRT during their follow up more than one year were older at first evaluation, had a higher body mass index (BMI) and lower FSH level at presentation, and experienced more frequently spontaneous puberty and resumption of their ovarian function.

**Table 2:** Comparison of women who stopped or continued their HRT.

	HRT stop <1 year n = 93	HRT stop > 1 year n = 69	p value
**Age at diagnosis (years)**	26.4 ±8.2	28.7 ±7.4	0.07
**Age at first evaluation (years)**	28.2 ±8.2	30.7 ±7.2	0.04
**Body mass index (kg/m**^**2**^**)**	21.7 ± 3.6	24.5 ±5.3	0.0002
**Spontaneous puberty**	78 (83.9%)	65 (94.2%)	0.043
**Primary amenorrhea**	23 (24.7%)	9 (13.0%)	0.064
**History of familial POI**	18 (19.6%)	18 (26.1%)	0.32
**Resumption of ovarian function during follow-up**	25 (26.9%)	30 (43.5%)	0.027
**Spontaneous pregnancy**	8/37 (21.6%)	7/39 (17.9%)	0.687
**Parental project**	41/83 (49.4%)	38/60 (63.3%)	0.098
**AMH at first evaluation (ng/ml)**	0.27±0.35	0.74±1.63	0.10
**FSH at first evaluation (IU/l)**	83.1 ± 44.6	64.2 ± 37.4	0.005
**Estradiol at first evaluation (pg/ml)**	24.6 ± 43.1	31.8 ± 44.2	0.30

Data are expressed as mean ±sd or number (%)

### Evaluation of bone mineral density

Ninety-two patients had bone mineral density (BMD) determination at initial evaluation and at B2. Clinical characteristics of these women are reported in Table 3. No patient was using bone antiresorptive, anabolic or bone remodeling medications, except Vitamin D treatment. At B1, 57 patients (62%) had normal BMD whereas 28 presented osteopenia and 7 had osteoporosis. At B2, 31 patients (34%) had normal BMD, 56 (61%) presented osteopenia, and 5 had osteoporosis (5%). In the whole cohort, loss of BMD during the follow up was evaluated at 41±140 mg/cm^2^ at vertebral level and at 32 ±720 mg/cm^2^ at femoral level. Thirty-one women (34%) had BMD impairment during follow up: 28 patients with initially normal BMD had progressed to osteopenia, none to osteoporosis and 3 patients with initial osteopenia had progressed to osteoporosis. Conversely, seven patients (8%) had BMD improvement during follow-up: 2 with osteopenia had normal BMD and 5 patients with osteoporosis had osteopenia. Among the 92 patients, 55 (60%) were on HRT at B2. Forty-two (46%) of these had stopped their HRT for over a year. Vitamin D status was determined in 89 patients and deficiency (<30 ng/ml) was diagnosed in 69 patients (77.5%). PTH levels were normal in all patients except 5 which present mild secondary hyperparathyroidism due to Vitamin D deficiency. Among them, 4 had normal BMD and one osteopenia. No patients had TSH levels above 10 mUI/L, and 5 between 5 to 10 mUI/L and none had hyperthyroidism history. No fractures were reported.

**Table 3:** Characteristics of POI women with two BMD evaluations, regarding their BMD status at the follow up evaluation.

BMD at B2	All results n = 92 (100%)	Normal n = 31 (34%)	Osteopenia n = 56 (61%)	Osteoporosis n = 5 (5%)	p value
**Age at B2 (years)**	40 [34–47]	40 [35–46]	40 [33–47]	42 [32–52]	0.82
**Age at POI diagnosis (years)**	28 [21–35]	28 [21–34]	28 [21–35]	18 [18–22]	0.22
**BMI (kg/m**^**2**^**)**	22 [20–26]	24 [21–28]^a^	22 [20–25]^a^	21 [20–22]	0.05
**Tobacco use**	27 (29.3%)	11 (35.5%)	15 (26.8%)	1 (20%)	0.63
**Use of HRT at B2**	55 (60%)	15 (48%)	36 (64%)	4 (80%)	0.26
**Vitamin D level (ng/ml)**	20 [13–29]	20 [14–29]	18 [13–27]	23 [22–30]	0.32

^a:^ p = 0.04

Data are expressed as median [interquartile interval] or number (%). B2: second evaluation

In univariate analysis, we found a significant loss of BMD in women who had stopped their HRT for over a year compared to women who did not: -57 mg/cm^**2**^ *versus* -13 mg/cm^**2**^ (p = 0.009) at femoral level. In vertebral level, we found a non-significant loss of BMD in women who had stopped their HRT for over a year compared to women who did not: -50 mg/cm^**2**^ *versus* -34 mg/cm^**2**^ (p = 0.60).

In multivariate analysis, after adjustment by stepwise model selection on age (p = 0.05), BMI (p = 0.48), smoking use (p = 0.22) and vitamin D deficiency (p = 0.69), and duration of POI (p = 0.003); discontinuation of HRT over one year was always associated with significant loss of femoral BMD: -17 mg/cm^**2**^ versus -52 mg/cm^**2**^ (p = 0.022). In vertebral level, we also found this non-significant trend -37 mg/cm^**2**^ versus -45 mg/cm^**2**^ (p = 0.80) without any significant variable.

There was also the reduction of BMD per year of HRT discontinuation from -33 mg/cm^**2**^ at femoral level to -41 mg/cm^**2**^ at vertebral level, whereas BMD gain per year of HRT was +23 mg/cm^**2**^ at femoral level to -19 mg/cm^**2**^ at vertebral level.

## Discussion

Very few studies focus on the following up women with primary POI or in the impact of HRT in these women. The current knowledge on POI comes from the greater part of observational studies integrating women with Turner syndrome [7,15]. This study is the first to analyse the long-term follow up and certain health status markers in women with POI. We clearly showed that HRT withdrawal during follow-up is common in this population, since it concerns 43% of our patients. We also found a high prevalence of BMD impairment, especially in patients who stopped their HRT.

An important finding of our study was the large number of patients stopping HRT and specialist care. This was attributed mostly to lack of interest or fear of breast cancer. Therefore, information concerning the absence of evidence that HRT increase breast cancer risk to a level greater than that found in normally menstruating women [16] have to be given to all POI women. Moreover, POI women have to be informed of objective benefice of HRT. Indeed, young women with untreated POI or early menopause are at increased risk of developing osteoporosis, along with cardiovascular disease and cognitive impairment [6,10,11,17–20]. In patients with Turner syndrome, an observational study showed that nearly 20% of patients did not receive HRT because of non-adherence [21]. We found that women who have stopped their HRT during their follow up had a lower FSH level at presentation, and experienced more frequently spontaneous puberty and resumption of their ovarian function. These women represented a subgroup of POI patients with higher probability of ovarian function resumption [22]. These events could therefore be the cause of HRT withdrawal by these women, since they wished to know exactly the status of their ovarian function. AMH was not different between the two groups: however, a main limitation of this findings is that different AMH assays were used between both evaluation, with different sensitivity; This had an influence AMH results, so further studies on AMH with sensitive assays should be conducted.

A very early menopause has a potentially devastating effect on long-term bone health [6,10,11,23,24]. Late menarche and menstrual irregularity are also been showed to be important risk factors for osteoporosis development [25]. In our study, we highlighted a reduction in bone mineral density during following up of POI women. We demonstrated that HRT discontinuation is the most important determinant of BMD in POI patients. The large number of patients in our cohort, a good capture rate (61%) of the study and the lack of significant differences in clinical presentation between POI patients with or without follow-up strengthens our results. The physiological bone mineral density loss depends on numerous factors, such as BMI, the delay between the evaluations, and tobacco use. In our study, after adjusting all confounding factors, discontinuation of HRT over one year was always associated with a significant loss of femoral BMD. Thus HRT impacts the reduction of bone mineral density in the population of patients with POI. Our results are consistent with a studies conducted showing that HRT induced a significant improvement in BMD in young POI women [26,27], and with a study on women with Turner syndrome, demonstrating a significant reduction of lumbar spine BMD in women not taking HRT [21]. In our study, none patients reported fracture in our study, so the role of HRT in the prevention of fracture have to be studied. Finally, we showed that other factors influencing BMD, as serum TSH and PTH, did not differ between women with or without BMD alteration, suggesting that these factors did not influence our results on BMD alterations. Furtheremore, our patients did not have history of other endocrine disease known to affect BMD, as hyperthyroidism or primary hyperparathyroidism.

The main limitation of our study is that we did not evaluate the whole cohort of POI patients, so there is a selective participation to our study. However, no differences concerning clinical and hormonal characteristics were found between the POI patients with or without follow-up. Our conclusions came from the largest published POI cohort analysis but have to be confirmed on another large and homogenous POI cohort. There is also a bias with the retrospective collection of data, based on self-reporting. Finally it is an observational study, without a control population. Nevertheless, the key point of our study is the importance of the cohort.

In conclusion, our study is the first follow-up analysis of a large cohort of patients with POI. Achieving improved care by physicians should then be expected to improve long-term outcomes for these patients, and precise information regarding HRT should be given, since compliance with this treatment has a direct impact on BMD. Prospective studies are needed to better describe the long-term impact of HRT in these patients, especially on prevention of osteoporotic fracture and on cardiovascular health. Finally, this finding highlights the need to encourage and not just assume that patients will take their HRT. We therefore have to promote specific therapeutic education for these patients, but also the healthcare professionals on this rare disease.
